# Six-month changes in ghrelin and glucagon-like peptide-1 with weight loss are unrelated to long-term weight regain in obese older adults

**DOI:** 10.1038/s41366-021-00754-0

**Published:** 2021-02-01

**Authors:** Jared J. Rejeski, Jason Fanning, Barbara J. Nicklas, W. Jack Rejeski

**Affiliations:** 1 Department of Internal Medicine, Section on Gastroenterology, Wake Forest School of Medicine, Winston-Salem, NC USA; 2 Department of Health & Exercise Science, Wake Forest University, Winston-Salem, NC USA; 3 Department of Internal Medicine, Section on Gerontology and Geriatric Medicine, Wake Forest School of Medicine, Winston-Salem, NC USA

**Keywords:** Obesity, Geriatrics, Obesity

## Abstract

**Background and objective:**

Weight loss (WL) and subsequent regain are complex physiologic processes, and our understanding of the hormonal changes associated with these processes continues to evolve. We aimed to examine the effects of behavioral WL on 6-month changes in ghrelin and GLP-1 and evaluate the effects of these changes in gut hormones on weight regain among older adults.

**Subjects and methods:**

One hundred seventy-seven obese (BMI: 33.5 (3.5) kg/m^2^) older adults (66.9 ± 4.7 years, 71.2% female, 67.6% white) were randomized to WL (WL; *n* = 68), WL plus aerobic training (*n* = 79), or WL plus resistance training (*n* = 75) for 18 months. Ghrelin, GLP-1, power of food scale (PFS), and weight were measured at baseline, 6 months, and 18 months.

**Results:**

There was no differential treatment effect on change in either gut hormone, however, there was a significant time effect across all groups (*p* < 0.001), with increases in ghrelin (∆ = +106.77 pg/ml; 95% CI = + 84.82, +128.71) and decreases in GLP-1 (∆ = −4.90 pM; 95% CI = −6.27, −3.51) at 6-month. Ratings on the PFS decreased from baseline to 6-month and there was significant loss of weight from baseline to either 6- or 18-month, ∆ = −7.96 kg; 95% CI = −7.95, −8.78 and ∆ = −7.80 kg; 95% CI = −8.93, −6.65, respectively (*p* < 0.001). Changes in ghrelin and GLP-1 at 6-month did not predict weight regain from 6- to 18-month.

**Discussion and conclusion:**

Among older adults with obesity and cardiometabolic disease, the intensive phase of dietary WL results in increasing levels of ghrelin and decreasing levels of GLP-1 that are unrelated to weight regain a year later. Registered with ClinicalTrials.gov (NCT01547182).

## Introduction

In a comprehensive review of the biological response to dieting, MacLean and colleagues [1] stated the following: “one of the primary outcomes from the neural adaptations to energy-restricted weight loss (WL) in obese individuals is an elevated appetite. This is more often detected in premeal or postabsorptive appetite sensations related to hunger, desire to eat, and prospective food consumption, but it has also been observed in postprandial and day-long assessments of these measures” (p. R585). From a biological perspective, two gut-related hormones that play a central role in appetite-related feelings and the regulation of body mass are ghrelin [2] and glucagon-like peptide-1 (GLP-1) [3]. More specifically, an increase in hedonic hunger (i.e., the drive to consume food in the absence of caloric deficit) is strongly related to increases in ghrelin [4] and decreases in GLP-1 [5]. In fact, in recent years, the study of GLP-1 agonists has been an active area of research in the pharmacological treatment of obesity [6]. Due to the absence of data on how ghrelin and GLP-1 respond to WL in older adults, the goal of the current investigation was to evaluate a change in ghrelin and GLP-1 as a result of a 6-month intensive behavioral WL intervention along with how these changes were related to a measure of hedonic hunger—the power of food scale (PFS) [7]. We then examine whether changes in these two gut hormones with WL are predictive of weight regain from 6- to 18-month.

Plasma levels of ghrelin tend to increase before and decrease following meals suggesting that it plays a role in the initiation of food consumption [8]. More relevant to the current investigation has been the observation that levels increase with WL [2]. Indeed, ghrelin is an orexigenic peptide and it may even play a role in the prevention of both skeletal muscle atrophy and neurodegenerative diseases [9]. In addition, elevated ghrelin levels have been implicated in Prader–Willi syndrome, a genetic condition characterized by excessive appetite and obesity [10], and may also be involved in the hedonic appeal of food given its effect on the mesolimbic dopaminergic system [11].

In contrast to ghrelin, GLP-1 has been found to decrease in response to WL [12]. GLP-1 affects appetite and weight maintenance in humans because it slows gastric emptying, reduces gut motility, and directly affects central regulation of appetite [3]. Research has found that interventricular injections of GLP-1 inhibit food intake, an effect that occurs independently of food in the stomach or the extent of gastric emptying [13]. Therefore, there are combined effects of GLP-1 on both the gut and brain that make it particularly important to appetite and the regulation of weight.

Our hypotheses were that fasting plasma levels of ghrelin would increase and levels of GLP-1 would decrease following a 6-month intensive behavioral WL intervention among older adults with obesity and cardiometabolic disease, effects that would be related to changes in weight during this time and to increases in hedonic hunger. Consistent with these patterns of change, we predicted that those experiencing the greatest change in these gut hormones would be at the greatest risk for weight gain from the end of the intensive phase of WL at 6-month to the final follow-up visit at 18-month.

## Methods

### Participants

The cooperative lifestyle intervention program-II (CLIP-II) study recruited both overweight and obese older adults with metabolic syndrome (MetS) and/or cardiovascular disease (CVD) into an 18-month randomized controlled trial (RCT) with three treatment groups: WL, WL plus aerobic training (WL + AT), or WL plus resistance training (WL + RT). The primary outcomes were changes in 400-m walking speed and strength. The methods have been described in detail [14]. In brief, delivery of this single-blinded RCT occurred in three community YMCAs in Forsyth County, NC, with the interventions being delivered by YMCA staff members. Eligible participants were community-dwelling men and women aged 60–79 years who were low active (i.e., engaging in <60 min/week of moderate to vigorous physical activity) with a body mass index ≥28 and <42 kg/m^2^, self-reported limitations in mobility, and documented evidence of CVD or an ATP II diagnosis of MetS [15]. We recruited individuals with CVD or MetS as these obesity-related conditions are highly prevalent in older adults [16]. Exclusion criteria included severe heart disease, severe systemic disease, having had a myocardial infarction or cardiovascular procedure in the previous 3 months, a blood glucose reading of ≥14 mg/dL, uncontrolled Type I or Type II diabetes, or a severe psychiatric condition. The institutional review board at Wake Forest School of Medicine approved the study protocol.

### Randomization

Recruitment occurred in eight waves with randomization of participants within each wave to one of the three interventions. Randomization occurred following baseline testing using a block randomization scheme that was stratified by a wave.

### Measures

Height was assessed without shoes to the nearest 0.25 cm using a stadiometer and body mass measured to the nearest 0.05 kg using a calibrated and certified digital scale. To determine levels of total ghrelin and GLP-1, blood samples (EDTA plasma) were collected in the early morning after a 12-h fast. All follow-up samples were collected at least 24 h after an exercise session and blood sampling was postponed (1–2 weeks after recovery of all symptoms) in the event of an acute respiratory, urinary tract, or other infection. Both ghrelin and GLP-1 assays were run in a single batch using enzyme-linked immunosorbent assay kits from MilliporeSigma (Burlington, MA). Ghrelin is assessed as picogram per milliliter (pg/mL) and GLP-1 is measured as a molar concentration (pM). Finally, the PFS assessed the drive to consume highly palatable foods in an obesogenic environment, with higher scores associated with the higher drive; it is a trait measure of hedonic appetite [7]. The measure includes subscale scores associated with three environments: food available, food present, and food tasted. For the purposes of this study, we utilized the total score of the PFS as it has good test–retest reliability (*r* = 0.77), internal consistency (*α* = 91), and construct validity [7, 17, 18].

### Intervention

The WL portion of the intervention comprised three phases: an *intensive* phase (months 1–6), a *transition* phase (months 7–12), and a *maintenance* phase (months 13–18). During the initial *intensive* phase, participants met with trained staff members at a local YMCA for three group sessions and one individual session each month. The group sessions, which lasted 60 min, were tapered to two monthly sessions and then one monthly session during the *transition* and *maintenance* phases, respectively. The session content was informed by the agency aspect of social cognitive theory [19], and the use of the group as an agent of change in the group dynamics literature [20, 21]. Specifically, CLIP-II employed a well-studied group-mediated cognitive-behavioral intervention with content focused on changing the determinants of self-efficacy (e.g., mastery experiences, social modeling) and setting highly specific proximal goals. The *intensive* phase of the study eased participants into diet and exercise goals to provide early mastery experiences and build self-efficacy. To facilitate week-to-week group discussions, the intervention included activities designed to foster small group formation. Self-regulatory skills were developed through weekly homework assignments, and support among members was encouraged through weekly discussions of successes, failures, and methods for overcoming barriers to individual and group progress. The *maintenance* phase transitioned participants away from staff- and group-supported self-regulation toward personal responsibility for self-regulation. Intermittent group discussions continued to provide opportunities for group member support. Initially, participants aimed to achieve a WL of 0.3 kg per week, with a distal goal of 7–10% reduction in body mass.

Regarding exercise in CLIP-II, individuals assigned to the WL + AT or WL + RT conditions engaged in four sessions of exercise training each week at one of the YMCAs. The AT prescription consisted primarily of walking on an indoor track 4 days each week, and participants were guided toward achieving 45 min of uninterrupted exercise at a rating of perceived exertion (RPE) of 12–14 on the Borg RPE scale [22] during each session. Those who received RT worked toward exercising for 45 min at an RPE of 15–18 on 4 days each week, completing exercises on eight Cybex resistance machines. During the first week, participants engaged in one set of 10–12 repetitions at 40% of their one-repetition max; a value determined during an orientation appointment. By weeks 3–12, the goal for participants was to complete three sets of 10–12 repetitions at 70% of their one-repetition max; a goal that was increased to 75% from week 13 onward. In addition, during this final phase, participants completed the third set to volitional fatigue. If they were able to achieve at least 12 repetitions, the resistance was increased to maintain a consistent RPE.

### Analyses

Descriptive statistics and box plots were initially computed on all variables to examine plots of the distributions for deviations from normality. An initial mixed model was run to examine the effects of treatment assignment and time of the assessment (baseline, 6-month, and 18-month) for both ghrelin and GLP-1. Because there was a significant time main effect, yet no treatment or time X treatment interaction, the analyses in this report focus on the changes in ghrelin and GLP-1 over time across treatment groups. Correlational analyses were then conducted to examine the relationship of changes in both ghrelin and GLP-1 with changes in weight, adjusting for baseline values. Finally, a linear model was conducted to examine the relationship between change in ghrelin and GLP-1 from baseline to 6 months to weight regain from 6- to 18-month. All analyses were conducted using SPSS Version 20; all authors had access to the study data and reviewed and approved the final manuscript.

## Results

### Participant characteristics

A CONSORT diagram and detailed recruitment and retention information have been published previously [23]. Of the 249 older adults randomized to treatment, 177 had blood collected at baseline and the 6th- and 18-month follow-up. The loss to follow-up did not differ by treatment group at either time point. Table 1 provides the baseline characteristics for the 177 participants, data that did not differ from the complete randomized sample [23]. The mean (SD) age of participants was 66.8 (4.6) years, and the baseline BMI was 34.0 (3.7) kg/m^2^. The sample was largely female (70.0%), 67.6% were white, and the educational background was diverse. Specifically, 41.8% had a high school diploma, and 56.0% had an associate degree or higher.

**Table 1:** Descriptive characteristics of participants (*n* = 177).

	Mean (SD) or Frequency (%)
Age in years	66.8 (4.6)
Female	124 (70.0%)
Race	
African American	53 (30.0%)
Hispanic	2 (0.9%)
White	118 (67.6%)
Other/mixed/missing	4 (1.8%)
Highest level of education	
Less than a high school diploma	4 (2.2%)
High school/some college	74 (41.8%)
Associate degree or higher	99 (56.0%)
400 M walk time (s)	332.7 (58.4)
BMI (kg/m^2^)	34.0 (3.7)
CVD history	30 (16.9%)
Diabetes	38 (21.5%)
Arthritis	110 (62.1%)
Hypertension	131 (74.0%)
Cancer	30 (16.9%)
Metabolic syndrome	148 (83.8%)

### Retention, adherence, and weight loss

Median (25th, 75th percentiles) attendance to scheduled treatment sessions was 71.1% (40.5, 83.3) for WL only, 83.1% (47.6, 92.9) for WL + AT, and 85.7% (70.7, 92.7) for WL + RT. All three groups lost significant weight from baseline to either the 6th (∆ = −7.96 kg; 95% CI = −7.95, −8.78) or 18th month (∆ = −7.80 kg; 95% CI = −8.93, −6.65) assessment. On average, there was no regain of weight from 6 months to 18 months (∆ = +0.16 kg, see means (95% CIs in Table 2)); however, there was considerable variability around the mean change in weight during this interval of time with an SD = 4.82 kg. Notable was the finding that participants’ sex had no effect on either WL or weight regain.

**Table 2:** Means (95% CIs) for study variables.

Time of assessment (*N* = 177)			
Variables	Baseline	6-Month	18-Month
Weight in kg	94.47 (92.24, 96.69)_a_	86.51 (84.36, 88.66)_b_	86.67 (84.40, 88.95)_b_
Ghrelin in pg/ml	529.50 (488.39, 570.61)_a_	636.27 (588.07, 684.46)_b_	600.21 (551.67, 648.73)_b_
GLP-1 p/M	29.26 (27.12, 31.39)_a_	24.36 (22.30, 26.43)_b_	25.21 (23.19, 27.24)_b_
Power of food	2.57 (2.43, 2.71)_a_	2.30 (2.17, 2.43)_b_	2.35 (2.22, 2.48)_b_

The means in rows not sharing a common subscript differ at *p* < 0.001.

### Change in ghrelin, GLP-1, and PFS scores from baseline to follow-up

Recall, that there were no treatment differences or treatment by time interactions for ghrelin, GLP-1, or PFS scores, however, as hypothesized, there were significant time effects (*p* < 0.001) from baseline to 6-month in levels of gut hormones with increases in ghrelin (∆ = +106.77 pg/ml; 95% CI = + 84.82, +128.71) and decreases in GLP-1 (∆ = −4.90 pM; 95% CI = −6.27, −3.51). Moreover, baseline to 18-month changes mirrored the effects observed from baseline to 6-month with a ∆ for ghrelin = +70.71 and ∆ = −4.05 for GLP-1; *p*-values < 0.001. In sensitivity analyses, we observed that neither baseline nor change in these two gut hormones was affected by baseline BMI nor comorbid conditions. In addition, mean PFS scores decreased (*p*-values < 0.001) from both baselines to 6 months (∆ = −0.28) and baseline to 18-month (∆ = −0.21). The mean (95% CIs) values for all measures at baseline, 6-month and 18-month can be found in Table 2.

### Change in gut hormones and change in weight from baseline to 6-month

Correlational analyses controlling for baseline values of variables in each analysis revealed that 6-month change in ghrelin was inversely related to 6-month WL (*r* = −0.44), whereas the 6-month change in GLP-1 was directly related to 6-month WL (*r* = 0.41); that is, greater short term WL was associated with increasing levels of ghrelin and decreasing levels of GLP-1. Interestingly, there were no statistically significant relationships (*p* > 0.05) between either baseline or 6-month change in PFS scores with 6-month changes in either gut hormone or body mass. To evaluate the independent contribution of each hormone to 6-month WL, we ran a linear regression. In combination, ghrelin and GLP-1 accounted for 30.2% of the variance in 6-month WL; both standardized beta weights were statistically significant (*p* < 0.001), *β* = −0.39 for ghrelin and +0.30 for GLP-1.

### Six-month change in ghrelin and GLP-1 and 18-month weight regain

In a final analysis, we examined whether the change in ghrelin and GLP-1 from baseline to 6 months was predictive of individual variation in weight regain from the 6-month to the 18-month assessment visit. Recall that, despite the expected changes in gut hormones following the intensive phase of treatment, there was no significant mean change in weight from 6- to 18-month; however, as noted previously, there was considerable variability in change in weight during this interval of time. A linear model, adjusting for baseline values yielded a nonsignificant *R*^2^ = 0.007.

## Discussion

The hormonal control of WL and regain is a complex process with both ghrelin and GLP-1 having been linked to both obesity [24] and changes in body weight over time [12]. These previously defined relationships have led to the development of GLP-1 agonists to successfully augment WL in obese patients, [25] including obese older adults [26]. Consistent with our hypotheses, we found that ghrelin increased and GLP-1 decreased following a 6-month intensive phase of behavioral WL therapy even though, contrary to expectations, participants' mean ratings on the PFS, a scale of hedonic hunger, declined. Although there are studies supporting the position that appetite increases with WL [27, 28], not all studies have found this to be true, [29] with evidence that several markers of appetite actually decrease with WL [30]. Interestingly, Maclean et al. [1] point out that elevated appetite with WL is often detected in premeal or postabsorptive sensations related to appetites, such as the desire to eat or craving. Perhaps a trait measure of hedonic hunger such as the PFS [7] is insensitive to the dynamic nature of variability in appetite across the day, an effect that has been supported by our own research related to the behavioral neuroscience of short-term food restraint [31, 32]. Elsewhere, we have argued that most existing research on behavior change has ignored the often rapid shifts that occur in response to changing physiological and psychological states throughout the day [33]. Thus, a definitive answer to whether changes in gut hormones relate to changes in appetite needs to be informed by studies that track appetite and hormonal responses across the day, but in particular to data collected before and following meals.

It was striking to find that the mean WL among participants in this study did not change from the sixth- to the 18-month assessment visit, despite the expected changes in gut hormones during the intensive phase of treatment. In fact, ghrelin and GLP-1 contributed independent information and explained 30.2% of the variance in WL at 6-month. Moreover, changes in ghrelin and GLP-1 from baseline to 18 months mirrored what was observed from baseline to 6 months and these changes remained significantly related to WL across the same interval of time. Clearly, as noted by others [3, 34], changes in gut hormones do track with both short- and long-term reductions in body mass [3, 34]. However, following a 6-month intensive WL intervention, changes in ghrelin and GLP-1 did not predict subsequent weight regain from 6 to 18 months. Although there was not a significant change in weight from 6- to 18-month, there was considerable variability around the mean change in weight with an SD = 4.82 kg. One possibility is that the strategies employed in state-of-the-art behavioral WL programs are adequate to counter the physiological pull exerted by elevation in ghrelin and decreases in GLP-1. Once participants are released from treatment, they relapse into previous habitual patterns of food consumption, wherein conditioned responses between the obesogenic environment and embodied cues rise again [35].

Because Barrea et al. [36] have observed in a sample of healthy adults that women were more adherent to a Mediterranean diet than men, we explored whether some of the variability in WL and/or weight regain in this study was due to participants’ sex. Our analyses failed to support a moderating role for sex. Of note, in a recent publication from our aging center [37], we conducted an integrative analysis across multiple WL studies of older adults (*N* = 1317). In this analysis, 6-month weight change achieved among those randomized to WL was −7.7%(95% CI −8.3% to −7.2%), with no difference noted by sex. Whereas men and women may differ in their response to a Mediterranean diet, they do not appear to differ in their response to traditional, behavioral WL therapy.

### Strengths and limitations

To our knowledge, this is the largest clinical trial of WL in older adults with obesity and cardiometabolic disease to track changes in ghrelin and GLP-1 during the intensive phase of treatment and to evaluate these changes as they relate to potential changes in hedonic hunger. Second, it is the first study to examine whether changes in ghrelin or GLP-1 following an intensive behavioral lifestyle intervention are predictive of weight regain 1-year later. There are several limitations to the study. First, the sample was largely older, Caucasian women, which did not permit us to examine whether there were differential responses based on race or sex. Second, we only collected data on gut hormones and body mass at baseline, 6-, and 18-month. And third, since we maintained contact with participants throughout the 18-month of the intervention and were unable to collect additional follow-up data, we could not determine whether these shifts in ghrelin and GLP-1 are determinants of weight regain following structured treatment for behavioral WL.

## Conclusions

Analysis of data from this large RCT found moderate relationships between WL following a 6-month intensive phase of treatment with increases in ghrelin and decreases in GLP-1. Changes in these hormones were neither related to a change in hedonic hunger nor to weight regain at an 18-month follow-up visit. Future research is warranted to examine how these hormones vary with appetite assessed across the day, particularly premeal and postabsorptive appetite sensations. In addition, studies should be conducted to determine whether changes in these hormones are related to weight regain following structured treatment for behavioral WL. In addition, it would be instructive to compare behavioral WL with behavioral WL coupled with a GLP-1 agonist.
